# Meta-analysis of genome-wide association studies uncovers shared candidate genes across breeds for pig fatness trait

**DOI:** 10.1186/s12864-022-09036-z

**Published:** 2022-11-30

**Authors:** Haonan Zeng, Zhanming Zhong, Zhiting Xu, Jinyan Teng, Chen Wei, Zitao Chen, Wenjing Zhang, Xiangdong Ding, Jiaqi Li, Zhe Zhang

**Affiliations:** 1 Guangdong Provincial Key Lab of Agro-animal Genomics and Molecular Breeding, National Engineering Research Center for Breeding Swine Industry, College of Animal Science, South China Agricultural University, 510642 Guangzhou, China; 2 Department of Animal Science, College of Animal Science, Zhejiang University, 310058 Hangzhou, China; 3 Key Laboratory of Animal Genetics and Breeding of the Ministry of Agriculture and Rural Affairs, National Engineering Laboratory for Animal Breeding, College of Animal Science and Technology, China Agricultural University, 100193 Beijing, China

**Keywords:** Pig, GWAS, meta-analysis, Backfat thickness, QTL

## Abstract

**Background:**

Average backfat thickness (BFT) is a critical complex trait in pig and an important indicator for fat deposition and lean rate. Usually, genome-wide association study **(**GWAS) was used to discover quantitative trait loci (QTLs) of BFT in a single population. However, the power of GWAS is limited by sample size in a single population. Alternatively, meta-analysis of GWAS (metaGWAS) is an attractive method to increase the statistical power by integrating data from multiple breeds and populations. The aim of this study is to identify shared genetic characterization of BFT across breeds in pigs via metaGWAS.

**Results:**

In this study, we performed metaGWAS on BFT using 15,353 pigs (5,143 Duroc, 7,275 Yorkshire, and 2,935 Landrace) from 19 populations. We detected 40 genome-wide significant SNPs (Bonferroni corrected *P* < 0.05) and defined five breed-shared QTLs in across-breed metaGWAS. Markers within the five QTL regions explained 7 ~ 9% additive genetic variance and showed strong heritability enrichment. Furthermore, by integrating information from multiple bioinformatics databases, we annotated 46 candidate genes located in the five QTLs. Among them, three important (*MC4R*, *PPARD*, and *SLC27A1*) and seven suggestive candidate genes (*PHLPP1*, *NUDT3*, *ILRUN*, *RELCH*, *KCNQ5*, *ITPR3*, and *U3*) were identified.

**Conclusion:**

QTLs and candidate genes underlying BFT across breeds were identified via metaGWAS from multiple populations. Our findings contribute to the understanding of the genetic architecture of BFT and the regulating mechanism underlying fat deposition in pigs.

**Supplementary Information:**

The online version contains supplementary material available at 10.1186/s12864-022-09036-z.

## Background

Human obesity has become an increasingly common social phenomenon in the past decades related to both quality of life and life expectancy [1]. Pig was often chosen as a model animal for scientific research due to its similar physiology architecture with human. Up to date, PigQTLdb (release 48) released 35,846 quantitative trait loci (QTLs), expression quantitative trait loci (eQTLs), and associations [2], of which 3,608 associations are related to fatness traits. Average backfat thickness (BFT) is an indicator for fat deposition and directly linked to fat content and lean rate in pig [3, 4]. Uncovering the genetic characteristics of BFT can be useful for pig genetic improvement.

Previous studies showed that BFT is a complex trait [5–7] with moderate or high heritability ranged from 0.2 ~ 0.6 [5–8]. Genome-wide association study (GWAS) in single population has been widely used to mine the potential QTLs and genes that associated with BFT [9–11]. And Gozalo‑Marcilla1 et al. [12] performed large scale GWAS of pig BFT from eight lines to highlight the genes involved in pathways for fat deposition. However, the power of single population GWAS is usually limited by sample size. Meta-analysis of GWAS summary statistics (metaGWAS) is an attractive method to increase the statistical power by integrating data from multiple breeds and populations. In recent years, metaGWAS has gradually become a popular method to study the genetic architecture of pig complex trait. For instance, Cai et al. [13] performed a large-scale metaGWAS in pig to identify the candidate gene of average daily gain across three breeds. Zhou et al. [14] conducted both GWAS and metaGWAS in pig to reveal new insights into the genetic architecture of average daily gain and lean meat percentage. Although, several candidate genes associated with BFT (e.g., *MC4R* [15, 16], *PPARD* [17], and *LEPR* [18, 19]) have been reported via GWAS and followed by experimental validations, the potential shared QTLs and genes of BFT across breeds are rarely reported.

The objectives of this study were to identify QTLs and candidate genes via metaGWAS and to explore the genetic architecture of BFT across pig breeds. In this study, we performed GWAS for BFT in 19 different populations genotyped with Porcine SNP BeadChip. Further, metaGWAS was performed for within-breed and across-breed strategies. Our findings contribute to the understanding of the genetic architecture of BFT and the regulating mechanism underlying fat deposition in pigs.

## Results

### Data summary and population stratification

The detail information (i.e., breed, sample size, genotypes, and phenotypes) of 19 populations used in this study were presented in Table 1. In total, we used data from 15,353 pigs consisting of 5,143 Duroc in seven populations, 7,275 Yorkshire in seven populations, and 2,935 Landrace in five populations. The average values (± standard deviation) of BFT for Duroc, Yorkshire, and Landrace were 10.87 (± 2.20 mm), 12.07 (± 3.36 mm), and 12.60 (± 4.28 mm), respectively.

**Table 1:** Summary of experimental data

Population	Breed	Number of Sample	Number of SNPs	Mean (mm)	Minimum (mm)	Maximum (mm)	SD (mm)	CV (%)	Chip
PP1	Duroc	1,993	39,311	9.82	4.58	17.45	1.66	16.94	a
PP2 [8]	Duroc	1,071	23,766	12.45	6.68	21.02	2.10	16.91	ab
PP3	Duroc	1,048	40,139	10.90	5.18	22.88	2.27	20.81	a
PP4	Duroc	353	39,082	12.47	8.98	16.59	1.45	11.64	b
PP5	Duroc	328	31,680	10.07	6.27	20.81	1.51	15.02	c
PP6	Duroc	190	39,927	12.07	8.06	19.71	2.02	16.70	a
PP7	Duroc	160	37,074	9.86	5.05	15.72	1.93	19.61	a
PP8	Yorkshire	2,179	41,314	11.14	3.02	23.35	2.51	22.55	ad
PP9	Yorkshire	1,794	41,360	10.74	4.29	29.09	2.73	25.41	ad
PP10	Yorkshire	1,259	45,002	11.77	5.03	22.01	2.42	20.56	a
PP11	Yorkshire	1,146	44,434	13.13	6.61	26.06	2.92	22.20	a
PP12	Yorkshire	406	44,200	16.25	7.17	22.76	2.36	14.51	a
PP13	Yorkshire	314	31,564	18.76	8.02	29.76	4.51	24.01	c
PP14	Yorkshire	177	40,070	12.02	7.53	17.33	1.66	13.83	c
PP15	Landrace	1,094	43,304	10.31	5.30	19.52	2.20	21.30	a
PP16	Landrace	556	30,705	18.89	8.66	29.69	4.51	23.86	c
PP17	Landrace	554	45,132	12.17	7.20	22.20	2.29	18.86	a
PP18	Landrace	552	43,644	12.00	5.75	22.87	2.71	22.59	a
PP19	Landrace	179	33,809	9.69	6.20	14.41	1.37	14.14	c

Chip a: GeneSeek GGP-Porcine Beadchip (Neogen Corporation, Lansing, MI, USA) with 50 K

Chip b: Illumina PorcineSNP60 BeadChip (Illumina, San Diego, CA, USA) with 60 K

Chip c: “Zhongxin­I” Porcine Breeding Chip (Beijing Compass Agritechnology Co., Ltd., Beijing, China) with 50 K

Chip d: GeneSeek GGP-Porcine Beadchip (Neogen Corporation, Lansing, MI, USA) with 80 K

**Fig. 1:**
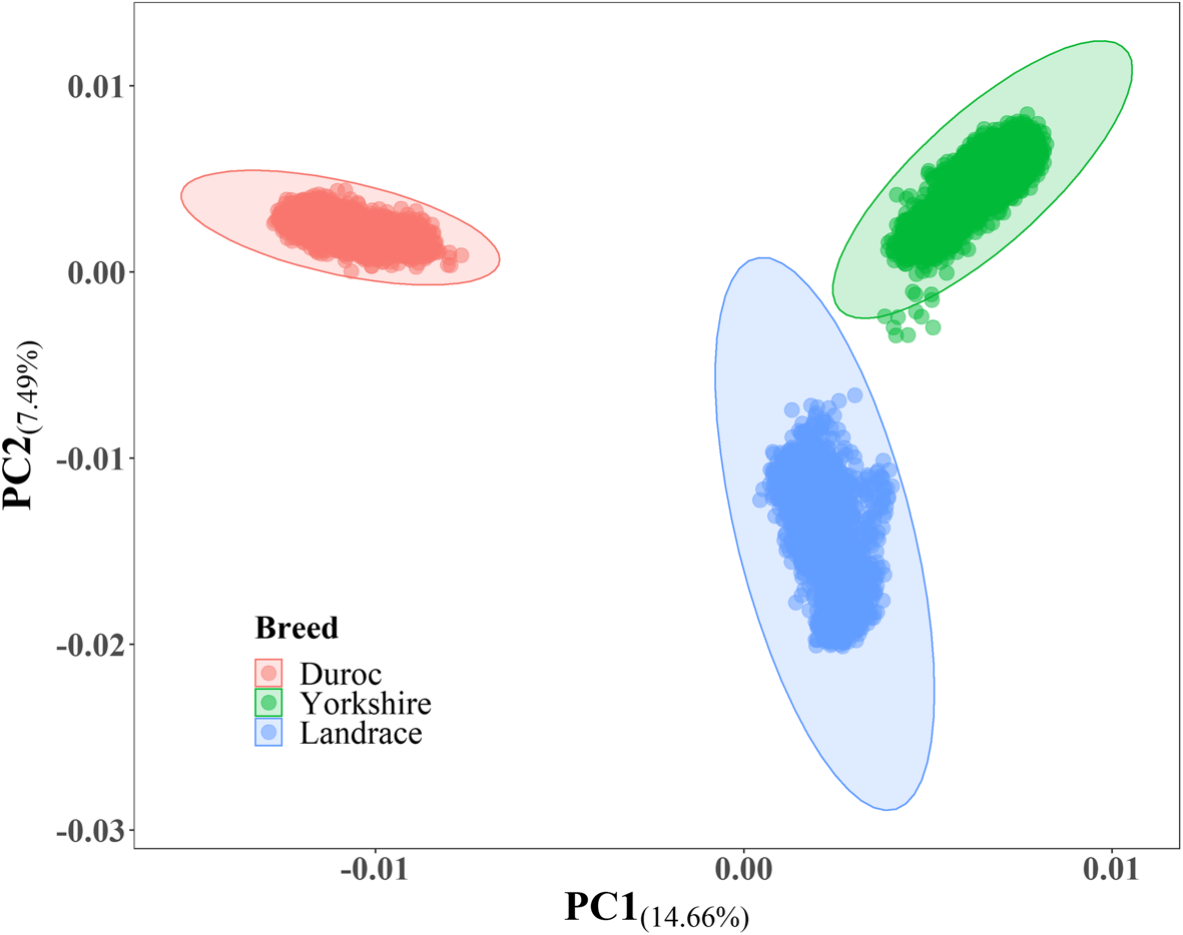
Population structure for nineteen populations. PC1 = the first principal component, PC2 = the second principal component. The number in brackets on axis represented the proportion of eigenvalues among all components

Principal component analysis (PCA) based on genotypes of all pigs (Fig. 1) and each of three breeds (Additional file 1: Figure S1) showed that samples from three breeds were clustered clearly. The first two genotype principal components (PCs) explained for 14.66% and 7.49% of total population variance, respectively.

### Meta‑analysis within breed

To overcome the limitation of power of single population GWAS, we then performed metaGWAS by integrating GWAS summaries from multiple populations with the same breed. As a basis for metaGWAS, we first conducted GWAS in each of 19 populations separately (Additional file 2: Table S1). In single population GWAS, the number of significant SNPs (*P* < 1 × 10^− 5^) ranged from 0 to 43. 105 SNPs were significant (*P* < 1 × 10^− 5^) only in one of 19 single population GWAS. 85 SNPs were significant (*P* < 1 × 10^− 5^) only in one of four metaGWAS (within-breed and across-breed), and 49 SNPs were significant in at least one metaGWAS. 28 SNPs were significant both in single population GWAS and metaGWAS (Additional file 3: Figure S2). The genomic inflation factor ($\lambda$) values for within-breed metaGWAS of Duroc, Yorkshire, and Landrace were 1.07, 1.05, 1.00, respectively (Table 2) and were within the normal range.

**Table 2:** Summary of QTLs from metaGWAS.

Breed	Lambda (λ)	Lead SNP	SSC	Position (bp)	-log_10_ (*P*-value)	QTL region	Number of significant SNPs within QTL in metaGWAS	Significant in single population GWAS (*P* < 1 × 10^− 5^)	Reported frequency in pigQTLdb
Duroc	1.07	rs81284646	1	161,824,864	7.70	155,986,286 ~ 161,824,864	10	Yes	21
		rs80936157	7	30,356,985	9.28	29,356,985 ~ 31,356,985	13	Yes	21
		rs81236473	18	10,555,467	6.29	9,555,467 ~ 11,555,467	1	No	1
Yorkshire	1.05	rs337892438	1	160,513,631	6.96	159,513,631 ~ 161,513,631	5	No	14
		rs81358998	2	49,246,142	6.86	48,246,142 ~ 50,246,142	2	Yes	0
Landrace	1.00	rs81359965	2	80,229,065	6.24	79,229,065 ~ 81,229,065	1	No	2
All breeds	1.07	rs319638368	1	53,666,889	6.80	52,666,889 ~ 54,666,889	20	No	0
		rs80877507	1	160,347,188	11.84	158,589,475 ~ 162,192,627	2	Yes	21
		rs81359652	2	60,697,443	7.56	59,697,443 ~ 61,697,443	1	No	2
		rs80962638	7	30,716,800	10.13	29,476,173 ~ 31,569,645	16	Yes	20
		rs81236473	18	10,555,467	8.16	9,555,467 ~ 11,555,467	1	No	1

**Fig. 2:**
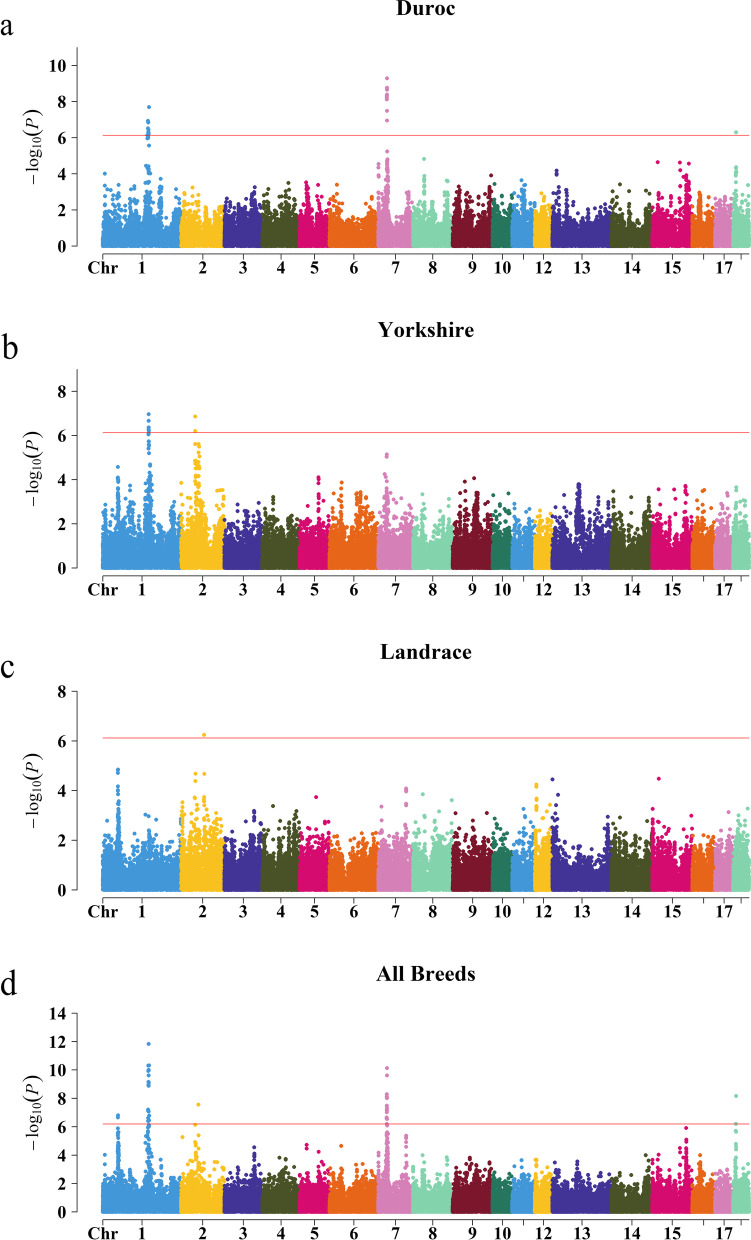
Manhattan plots of metaGWAS within breed (**a, b, c**) and across breeds (**d**). The red lines indicate the 5% genome-wide Bonferroni corrected thresholds as -log_10_*P* equal to 6.14, 6.14, 6.12, and 6.20, respectively

In Duroc within-breed metaGWAS, a total of 24 significant SNPs were detected (Additional file 4: Table S2). Further, we identified three QTLs on *sus scrofa* chromosome (SSC) 1, 7, and 18 respectively (Fig. 2a, Additional file 5: Figure S3a). The QTL on SSC7 was identified by the most significant lead SNP *rs80936157* (7:30356985, -log_10_*P* = 9.28) which was located in the gene body of *NUDT3*. The QTL on SSC1 included the second most significant lead SNP *rs81284646* (1:161824864, -log_10_*P* = 7.70) which was located in the gene body of ENSSSCG00000004911. And the QTL on SSC18 was detected via the lead SNP (18:10555467, -log_10_*P* = 6.29) which was located in the gene body of *ZC3HAV1*.

In Yorkshire within-breed metaGWAS, we identified seven significant SNPs (Additional file 4: Table S2) and discovered two QTLs on SSC1 and SSC2, respectively (Fig. 2b, Additional file 5: Figure S3b). The QTL on SSC1 was defined by the most significant lead SNP *rs337892438* (1:160513631, -log_10_*P* = 6.96) which was located in the downstream of *ENSSSCG00000045579* with distance equal to 69,671 bp. The QTL on SSC2 included the second most significant lead SNP *rs81358998* (2:49246142, -log_10_*P* = 6.86) which was located in the gene body of *SBF2*.

In Landrace within-breed metaGWAS, we only identified one significant SNP (Additional file 4: Table S2) and one QTL on SSC2 (Fig. 2c, Additional file 5: Figure S3c). The lead SNP *rs81359965* (2:80229065, -log_10_*P* = 6.24) was located in the gene body of *RMND5B*.

### Meta‑analysis across breeds

To maximize the sample size to discover the significant QTLs associated with BFT, we conducted an across-breed metaGWAS combining GWAS summary statistics of all 19 populations. The inflation factor ($\lambda$) value was 1.07 for this across-breed metaGWAS (Table 2). A total of 40 significant SNPs (Additional file 4: Table S2) and five QTLs on SSC1, 2, 7, and 18 (Fig. 2d, Additional file 5: Figure S3d) were detected.

Five QTLs were detected in across-breed metaGWAS. Two out of the five QTLs (SSC1, 52,666,889 ~ 54,666,889 bp; SSC2, 59,697,443 ~ 61,697,443 bp) were not observed in within-breed metaGWAS, while the remaining three QTLs were overlapped with those in within-breed metaGWAS.

The QTL on SSC1 was defined by the most significant lead SNP *rs80877507* (1:160347188, -log_10_*P* = 11.84) which was located in the gene body of *ENSSSCG00000048538*. The QTL on SSC7 was identified by the second most significant lead SNP *rs80962638* (7:30716800, -log_10_*P* = 10.13) which was located in the gene body of *SNRPC*. The QTL on SSC18 included the third most significant lead SNP *rs81236473* (18:10555467, -log_10_*P* = 8.16) which was located in the gene body of *ZC3HAV1*. The QTL on SSC2 included the lead SNP *rs81359652* (2:60697443, -log_10_*P* = 7.56) which was located in the downstream of *ENSSSCG00000013869* with distance equal to 14,538 bp. The remaining QTL was detected on SSC1 by the lead SNP (1:53666889, -log_10_*P* = 6.80) which was located in the downstream of *CEP162* with distance equal to 224,490 bp.

We also examined the linkage disequilibrium (LD) blocks across breed for each of the five QTLs (Additional file 6: Figure S4). And the two QTLs located on SSC1 showed high regional LD while the QTL located on SSC18 showed the low LD. Obviously, the LD pattern showed a high similarity across breeds.

### Post‑GWAS analysis

Genomic heritability estimation and predictive ability of five across-breed QTLs.

**Fig. 3:**
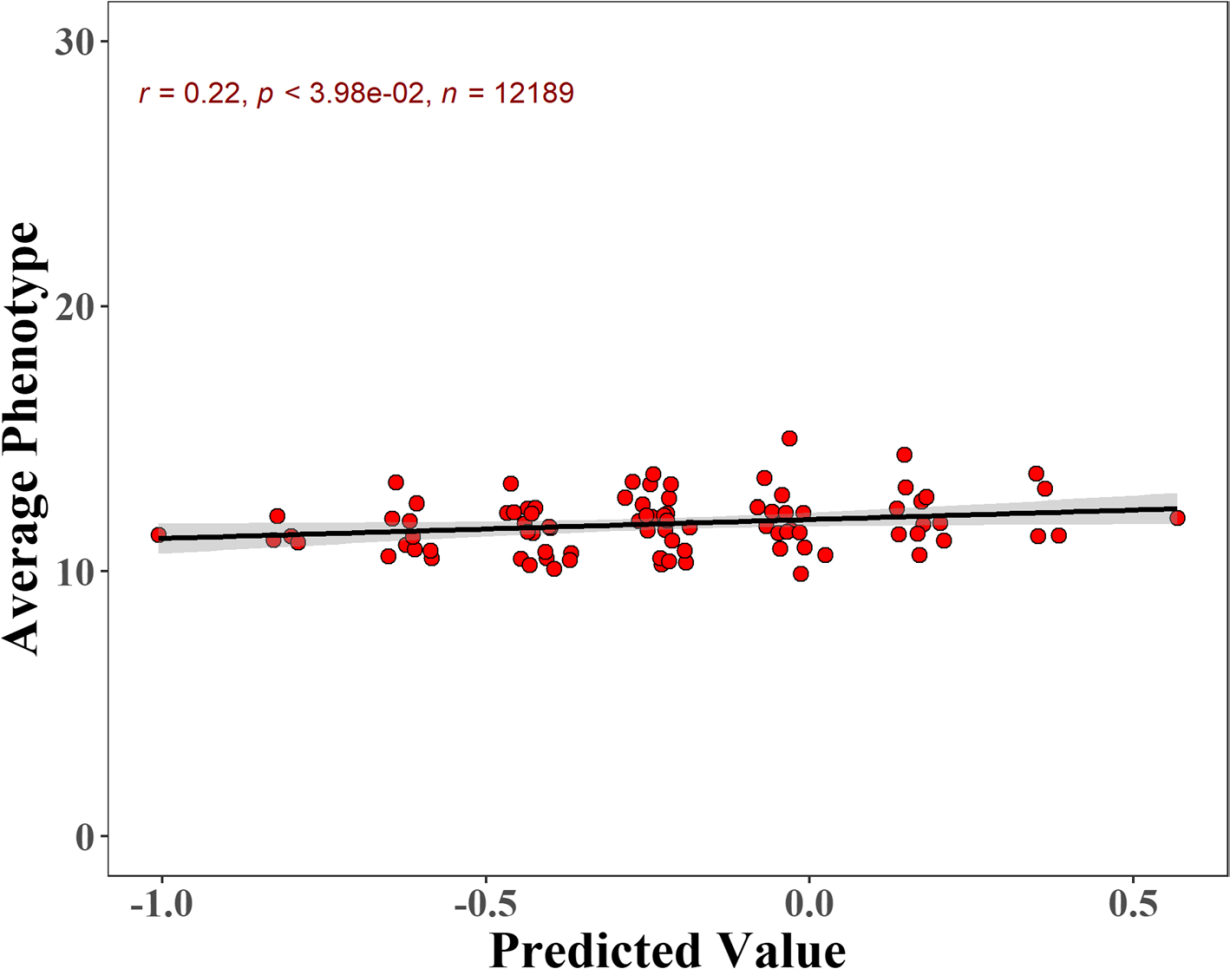
The correlation between predicted values and average phenotypes. Each point represented one genotype combination in five lead SNPs. Shaded part represented the 95% confidence interval

To verify the reliability of metaGWAS results, we conducted heritability estimation (Table 3) and predictive ability evaluation (Fig. 3). Given that the standard error of heritability estimates is influenced by sample size, we used the largest population in each breed for heritability estimation to obtain a reliable estimate of heritability.

**Table 3:** Estimated heritability of the five QTLs in across-breed metaGWAS.

Population	Breed	Number of SNPs	${\varvec{h}}_{{QTLs}}^{2}$ (SE)	${\varvec{h}}_{{genome}}^{2}$ (SE)	Proportion ($\upsigma _{{QTLs}}^{2}$/$\upsigma _{{genome}}^{2}$)	Enrichment
PP1	Duroc	157	0.01(0.01)	0.20(0.03)	0.07	18.15
PP8	Yorkshire	169	0.02(0.01)	0.21(0.03)	0.09	21.63
PP15	Landrace	174	0.02(0.02)	0.22(0.05)	0.09	22.43

${\varvec{h}}_{{QTLs}}^{2}$: Estimated heritability of SNPs in the five QTLs in our study; ${\varvec{h}}_{{genome}}^{2}$: Estimated heritability of all SNPs; $\upsigma _{{QTLs}}^{2}$: Genetic variance of SNPs in the five QTLs in our study; $\upsigma _{{genome}}^{2}$: Genetic variance of all SNPs

The estimated heritability for SNPs within the five QTL regions was 0.01 for Duroc, 0.02 for Yorkshire, and 0.02 for Landrace. These SNPs explained 7.25%, 8.85% and 9.01% additive genetic variance for Duroc, Yorkshire, and Landrace respectively. It should be noted that the five QTL regions were 18.15, 21.63, and 22.43 folds enriched for heritability in Duroc, Yorkshire, and landrace, respectively.

To evaluate whether the five QTLs are effective across-breed, we conducted genomic prediction with the lead SNPs within these QTLs, and calculated the Pearson’s correlation coefficient between predicted genetic value and average phenotype value in all populations. Finally, we observed an significant predicting ability across breed for these QTLs (Pearson’s correlation coefficient = 0.22, *P* < 3.98 × 10^− 2^).

#### Candidate genes mapping

To investigate the gene that potentially regulates BFT, we conducted candidate gene mapping from total 138 positional candidate genes via four external biological annotations (Table 4, Additional file 7: Table S3). Within the five QTLs, we identified 46 candidate genes based on functional annotations in Gene Ontology (GO) and Kyoto Encyclopedia of Genes and Genomes (KEGG), PigQTLdb and NHGRI-EBI. Several genes were identified in three annotations such as *MC4R* (SSC1, ~ 160.8 Mb), *PPARD* (SSC7, ~ 31.2 Mb), *RELCH* (SSC1, ~ 159.2 Mb), and *ITPR3* (SSC7, ~ 29.8 Mb), of which *MC4R* was widely reported in both pig (11 evidence in backfat) and human (24 evidence in body mass index). *SLC27A1* (SSC2, ~ 60.2 Mb) was the gene annotated in five fat metabolism processes in GO and KEGG including lipid, fatty acid, and triglyceride metabolic pathways, insulin signaling pathway and homeostasis regulation pathway. Two genes, *ILRUN* (SSC7 at ~ 30.6 Mb) and *KCNQ5* (SSC1 at ~ 52.4 Mb) were not previously reported in pig but reported in human research.

**Table 4:** Candidate genes in the five QTLs in across-breed metaGWAS

SSC	QTL Position	Candidate Genes
1	52,666,889 ~ 54,666,889	*KCNQ5* ^c^, *CYB5R4*^c^, *CEP162*^c^, *NT5E*^b^
1	155,986,286 ~ 161,824,864	*PHLPP1* ^bc^, *RELCH*^abc^, *RNF152*^b^, *CDH20*^b^, *MC4R*^abc^, *CCBE1*^b^, *GRP*^c^, *SEC11C*^c^, *ALPK2*^b^
2	59,697,443 ~ 61,697,443	*MAST3* ^c^, *JAK3*^a^, *B3GNT3*^a^, *SLC27A1*^a^, *NXNL1*^a^, *U3*^c^, *KLF2*^a^, *KCNN1*^a^
7	29,476,173 ~ 31,569,645	*B3GALT4* ^a^, *ITPR3*^abc^, *IP6K3*^a^, *LEMD2*^c^, *GRM4*^c^, *NUDT3*^c^, *PACSIN1*^c^, *SPDEF*^c^, *ILRUN*^c^, *SNRPC*^c^, *UHRF1BP1*^c^, *ANKS1A*^c^, *TCP11*^c^, *SCUBE3*^c^, *PPARD*^abc^, *TEAD3*^c^, *TULP1*^c^, *FKBP5*^c^
18	9,555,467 ~ 11,555,467	*KDM7A* ^c^, *TBXAS1*^a^, *ZC3HAV1*^c^, *TRIM24*^c^, *AKR1D1*^a^, *CREB3L2*^ab^, *FMC1*^a^

Superscript numbers: ^a^GO/KEGG annotation; ^b^PigQTLdb annotation; ^c^NHGRI-EBI annotation

## Discussion

In this research, we conducted metaGWAS for BFT by combining 19 populations across three commercial breeds and identified shared QTLs across breeds. The BFT is a complex trait with heritability of 0.20 ~ 0.22 in our study, which was agreed with previous studies [7, 8, 20–22]. On one hand, additive genetics variance explained by SNPs among the defined QTL regions ranged from 7.25 to 9.01% for the three breeds. These SNPs also showed high enrichment in heritability for all breeds, which indicated a shared genetic architecture of BFT across breeds at these QTL regions. On the other hand, across-breed metaGWAS showed a greater power to detect potential QTLs compared with within-breed metaGWAS. For instance, QTLs detected by across-breed metaGWAS on SSC1 (~ 160 Mb) and SSC7 were significant in both Duroc and Yorkshire within-breed metaGWAS, but not significant in Landrace. Similarly, QTLs detected by across-breed metaGWAS on SSC1 (~ 50 Mb) were significant in both Yorkshire and Landrace within-breed metaGWAS, but not significant in Duroc. Finally, five QTLs were identified with a high enrichment of heritability and a moderate predictive ability for across breeds. These results indicate that across-breed metaGWAS could identify shared QTLs for BFT across breeds.

Analysis method is crucial for association study, as complicated population structure and relatedness have the potential to lead to false signals [23–25]. Recent studies have also shown that inclusion of candidate maker in the SNP-derived genetic relationship matrix (GRM) can lead to a loss of power [26–28]. Therefore, we used GCTA-LOCO for single locus regression in order to avoid “proximal contamination” [27, 29] and corrected population stratification and relatedness by the mixed linear model and the first five PCs [30–33]. In addition, considering the data inconsistency among each population, we unified them into summary statistics independently by single population GWAS. Then we used summary statistics to performed metaGWAS to enhance the power of detecting significant SNPs.

Among the discovered candidate genes, three genes associated with BFT were well studied. First, *MC4R* (SSC1, ~ 160 Mb) was associated with fatness, growth, as well as meat quality traits reported in recent decades [12, 15, 34–36]. The reliability of *MC4R* had been validated by several studies in pigs [37–39] and in human [40–42]. In this study, *MC4R* was located in the QTL on SSC1 (~ 160 Mb) and was annotated with several homeostasis regulation pathways in GO biological processes. Second, *PPARD* is recognized as an important gene associated with fat deposition traits in gene and gene expression layers [17, 43, 44], which was detected in our study in QTL on SSC7 and annotated by all the databases. Third, *SLC27A1* was another detected important genes that showed multiple evidence associated with fat deposition [45–47]. In addition, we identified seven promising candidate genes, *PHLPP1*, *NUDT3*, *ILRUN*, *RELCH*, *KCNQ5*, *ITPR3*, and *U3*. *RELCH*, the candidate gene near *MC4R*, belonged to sterol and lipid metabolism pathway in GO database and was also identified in previous researches [14, 48]. *ITPR3*, the candidate gene near *PPARD*, belonged to insulin secretion pathway in KEGG database and both annotated in pig [49] and human. *PHLPP1* (SSC1, ~ 160 Mb) and *NUDT3* (SSC7), two candidate genes near major genes *MC4R* and *PPARD* respectively, were found to be associated with backfat in pig [8, 14, 50–53]. Three of them, *ILRUN*, *KCNQ5*, and *U3* were reported in human associations studies in BMI and body weight for 15, 7, and 9 times respectively but not reported as the candidate genes in pig.

The sample size is a crucial factor to the power of GWAS. Generally, there are two strategies to enlarge the sample size. One is to combine individual level data (genotypes and phenotypes) from multiple populations, then to conduct GWAS analysis. The other is metaGWAS, in which the GWAS summary statistics from multiple populations are analyzed jointly, rather than put individual data together. In this study, we aimed at testing the metaGWAS strategy, in which individual data from different populations are not allowed to be combined together. The other strategy would be an interesting solution deserve further attempt in our further investigation. Integrating data from multiple populations could further increase the power of association analysis. However, the weakness of metaGWAS still could not be neglected. For example, the available SNPs were different for different populations, hence the number of common SNPs across all populations were usually too small to perform GWAS effectively. Therefore, a large pig genotype imputation reference panel was necessary for unifying the SNP maps for different populations.

## Conclusion

In this study, we conducted metaGWAS for BFT on 15,353 pigs from diverse genetic backgrounds. We identified 40 genome-wide significant SNPs located in five QTL regions and annotated 46 candidate genes based on across-breed metaGWAS. Among the candidate genes, *MC4R*, *PPARD*, and *SLC27A1*, had been well studied. Additionally, seven candidate genes, *PHLPP1*, *NUDT3*, *ILRUN*, *RELCH*, *KCNQ5*, *ITPR3*, and *U3*, were identified to be promising candidate genes associated with BFT. Our results provided useful reference for understanding the genetic architecture of BFT and the regulating mechanism underlying fat deposition in pigs.

## Materials and methods

### Population and data

A total of 15,353 pigs (5,143 Duroc, 7,275 Yorkshire, and 2,935 Landrace) from 19 populations in 12 Chinese pig farms were used in this analysis. The average age of pigs was 156, 172, and 164 days for Duroc, Yorkshire, and Landrace respectively. The backfat thickness was measured by living B-ultrasonic within each population at the end of test. The measurements were adjusted to body weight at 100 kg using within farm genetic evaluation system. Details about the populations, phenotypes, and genotypes were shown in Table 1.

All pigs were genotyped with one of the following SNP chips, GeneSeek GGP-Porcine Beadchip 50 and 80 K (Neogen Corporation, Lansing, MI, USA), Illumina PorcineSNP60 BeadChip 60 K (Illumina, San Diego, CA, USA), “Zhongxin­I” Porcine Breeding Chip 50 K (Beijing Compass Agritechnology Co., Ltd., Beijing, China). The annotation of all SNPs has been unified to Ensemble *Sus scrofa* 11.1 reference genome version. And the missing genotypes were phased within each population using Beagle 5.1 [54]. Quality control on genotypes were carried out within each population using PLINK v1.9 [55] with the criterion of minor allele frequency > 0.01 and *P* > 1 × 10^− 6^ for Hardy-Weinberg equilibrium test. SNPs with no position information or located on sex SSC were removed. After quality control, the number of SNPs within each population was shown in Table 1.

### Population structure analysis

To correct the population structure in GWAS model and investigate the population structure, we performed PCA using GCTA-PCA (1.94.0beta) [30, 56] in single population and mixed population, respectively. After that we visualized the result using R package ggplot2 [57].

### Single population GWAS

We performed GWAS in each single population separately using GCTA-LOCO [29, 56] with the following mixed linear model:

$${\varvec{y}}={{\varvec{X}}_{{\varvec{s}}{\varvec{n}}{\varvec{p}}}}{{\varvec{b}}_{{\varvec{s}}{\varvec{n}}{\varvec{p}}}}+{{\varvec{X}}_{\varvec{c}}}{{\varvec{b}}_{\varvec{c}}}+{{\varvec{g}}^ - }+{\varvec{e}}$$

where ${\varvec{y}}$ is an $n \times 1$ vector of phenotypic values; ${{\varvec{X}}_{{\varvec{s}}{\varvec{n}}{\varvec{p}}}}$ is the SNP genotype indicator variable coded as 0, 1 or 2 with its additive effect ${{\varvec{b}}_{{\varvec{s}}{\varvec{n}}{\varvec{p}}}}$; ${{\varvec{X}}_{\varvec{c}}}$ is the incidence matrix of fixed covariates (test farm, test year, test season, sex and the first five principal components) with their corresponding coefficient ${{\varvec{b}}_{\varvec{c}}}$; ${{\varvec{g}}^ - }$ is the accumulated genetics effect captured by all SNPs except those on the chromosome where the candidate SNP is located via ${{\varvec{g}}^ - }\sim N\left( {0,{\varvec{G}}\sigma _{g}^{2}} \right)$, where ${\varvec{G}}$ is the SNP-derived genetic relationship matrix follows the formula [58]: ${\varvec{G}}=\frac{{{\varvec{Z}}{\varvec{Z}}^{\prime}}}{{2\sum {p_i}\left( {1 - {p_i}} \right)}}$, where ${\varvec{Z}}$ was the minor allele frequency (MAF) adjusted genotype matrix with elements ($0 - 2{p_j}$), ($1 - 2{p_j}$), and ($2 - 2{p_j}$); ${\varvec{e}}$ is a vector of residuals with ${\varvec{e}}\sim N\left( {0,{\varvec{I}}\sigma _{e}^{2}} \right)$. The variance-covariance matrix of ${\varvec{y}}$ is ${\varvec{V}}={\varvec{G}}\sigma _{g}^{2}+{\varvec{I}}\sigma _{e}^{2}$.

### Meta-analysis of GWAS

To perform meta-analysis of GWAS, we utilized the GWAS summary statistics from single population GWAS as input for METAL (released on 2011-03-25) [59], in which inverse-variance weighted fixed-effects model was implemented with parameters “SCHEME STDERR” and “GENOMICCONTROL ON”. We set the genome-wide significant threshold of metaGWAS via Bonferroni correction (0.05/number of SNPs). The Manhattan plots and Quantile-Quantile (Q-Q) plots as well as $\lambda$ [60] were performed using R program. To detect the QTLs within and across breed, we performed metaGWAS with GWAS summary statistics from a single breed or all breeds, which were defined as within-breed and across-breed metaGWAS, separately. To fairly compare single population GWAS with metaGWAS, we used the same genome-wide significance level (1 × 10^− 5^) for both method in the comparative analysis.

### Definition of QTL and LD analysis

In this study, we defined QTL as the genomic region containing a set of significant SNPs, where physical distance of each neighbor pair was less than 5 Mb. If the length of a QTL region was less than 2 Mb, the region was defined as the 1 Mb on the either side of the lead SNP. A lead SNP was defined as the most significant SNP within a QTL.

To assess the LD event in each QTLs across breeds, we computed the coefficient of LD (r^2^) in the largest population within each breed (PP1, PP8, PP15) using PLINK v1.9 [55], and displayed LD blocks using R package LDheatmap [61]. In addition, to compare the result in this study with that in previous studies, we summarized each QTL overlapped frequency between this study and pigQTLdb on 25 March 2022 [2] with trait “backfat” and distance shorter than 5 Mb.

### Estimation of genomic heritability explained by SNPs

To estimate the genomic heritability explained by SNPs within identified QTLs, SNPs were partitioned into two sets, within QTLs and outside QTLs. Then they were imported as two variance components in mixed linear model to estimate heritability using GCTA-GREML [56, 62]:

$${\varvec{y}}={\varvec{X}}{\varvec{b}}+{{\varvec{Z}}_{within}}{{\varvec{u}}_{within}}+{{\varvec{Z}}_{outside}}{{\varvec{u}}_{outside}}+{\varvec{e}}$$

where ${\varvec{y}}$ is an $n \times 1$ vector of phenotypic values; ${\varvec{X}}$ is the incidence matrix of fixed covariates (test farm, test year, test season and the first five PCs) with their corresponding coefficient ${\varvec{b}}$; ${{\varvec{u}}_{within}}$ and ${{\varvec{u}}_{outside}}$ represent additive effects explained by the SNPs within or outside all QTL regions via ${\varvec{u}}\sim N\left( {0,{\varvec{G}}\sigma _{g}^{2}} \right)$, where ${\varvec{G}}$ is the SNP-derived genetic relationship matrix follows the formula [58]: ${\varvec{G}}=\frac{{{\varvec{Z}}{\varvec{Z}}^{\prime}}}{{2\sum {p_i}\left( {1 - {p_i}} \right)}}$, where ${\varvec{Z}}$ was the minor allele frequency (MAF) adjusted genotype matrix with elements ($0 - 2{p_j}$), ($1 - 2{p_j}$), and ($2 - 2{p_j}$).${{\varvec{Z}}_{within}}$ and ${{\varvec{Z}}_{outside}}$represented incidence matrix explained by the SNPs within or outside identified QTLs. ${\varvec{e}}$ is a vector of residuals with ${\varvec{e}}\sim N\left( {0,{\varvec{I}}\sigma _{e}^{2}} \right)$. The heritability proportion for the SNPs within QTLs was defined as $\frac{{\upsigma _{{within}}^{2}}}{{\upsigma _{{within}}^{2}+\upsigma _{{outside}}^{2}}}$.

### Predictive ability of genotype combination of five lead SNPs across populations

To evaluate the predictive ability, we computed prediction value of phenotype from five lead SNPs (detected in across-breed metaGWAS) genotype combinations via the formula [21]:

$${{\varvec{y}}_{prediction}}=\mathop \sum \nolimits_{{i=1}}^{5} {\hat {\varvec{\beta}}_{\varvec{i}}}{{\varvec{G}}_{\varvec{i}}}{\text{~}}$$

where ${{\varvec{y}}_{prediction}}$was the prediction value of phenotype; ${\hat {\varvec{\beta}}_{\varvec{i}}}$ denotes the estimate of marginal genetic effect in association summary statistics; ${{\varvec{G}}_{\varvec{i}}}$ denotes the number of effect alleles for each genetic variant coded as 0, 1, or 2. The Person’s correlation between predicted values and average phenotypes every genotype combination (sample size > 30) was calculated as predictive ability.

### Identification candidate genes by functional annotation

To identify the candidate genes from QTLs, we firstly retrieved them based on physical location (within QTLs detected in across-breed metaGWAS) and multi external biological annotations. Second, we matched genes within QTLs with those belong to fat regulation biology process (“lipid/fatty acid/sterol/triglyceride/bile acid metabolic pathways”, “insulin signal pathways”, “homeostasis regulation pathways”) in GO [63, 64] and KEGG [65, 66]. In this step, enrichment test was calculated based on hypergeometric distribution using R-package clusterProfiler [67] with commands “enrichGO” and “enrichKEGG”. Third, we matched genes within QTLs with those associated with pig traits (“backfat”, “obesity index”, “body weight”, “body mass index”, “intramuscular fat content”, lean meat percentage”, “feed intake”) in pigQTLdb [2]. Finally, we matched genes within QTLs with whose associated with human obesity-related traits (“body mass index”, “body weight”, “obesity”, “energy intake”) in NHGRI-EBI Database [68].

## Supplementary Information

**Additional file 1:** **Figure S1.** PCA plot of each of three breeds.

**Additional file 2:** **Table S1.** Summary information of 19 single population GWAS.

**Additional file 3:** **Figure S2.** Venn diagram of the number of significant SNPs (*P*<10^-5^) between 19 single population GWAS and four metaGWAS.

**Additional file 4:** **Table S2.** Summary information of significant SNPs in four metaGWAS.

**Additional file 5:** **Figure S3.** Q-Q plot of  four metaGWAS.

**Additional file 6:** **Figure S4.** LD blocks of each of the five QTLs in three breeds.

**Additional file 7:** **Table S3 (Sheet1).** Annotation of candidate genes belongs to fat regulation biology process in GO and KEGG. **Table S3 (Sheet2).** Annotation of candidate genes in pigQTLdb. **Table S3 (Sheet3).** Annotation of candidate genes in human NHGRI-EBI Catalog.

## Data Availability

All annotation information was obtained from a publicly available source (http://​www.​ensem​bl.​org). The genotype and phenotype used and/or analyzed during the current study are available from the corresponding author on reasonable request.

## Abbreviations

BFT: average backfat thickness
CV: coefficient of variation
eQTL: expression quantitative trait locus
GO: Gene Ontology
GRM: SNP-derived genetic relationship matrix
GWAS: genome-wide association study
KEGG: Kyoto Encyclopedia of Genes and Genomes
LD: linkage disequilibrium
LOCO: leave-one-chromosome-out
metaGWAS: meta-analysis of genome-wide association studies
MLM: mixed linear model
PCA: principal component analysis
PC: principal component
QTL: quantitative trait locus
SD: standard deviation
SE: standard error
SNP: single-nucleotide polymorphism
SSC: *sus scrofa* chromosome
λ: genomic inflation factor (lambda)
