# Supplementary material for: Meta-analysis of genome-wide association studies uncovers shared candidate genes across breeds for pig fatness trait
Source: BMC Genomics. 2022 Nov 30;23:786. doi: 10.1186/s12864-022-09036-z (PMC9714057; doi:10.1186/s12864-022-09036-z)

Additional file 1: Figure S1a. PCA plot of Duroc.

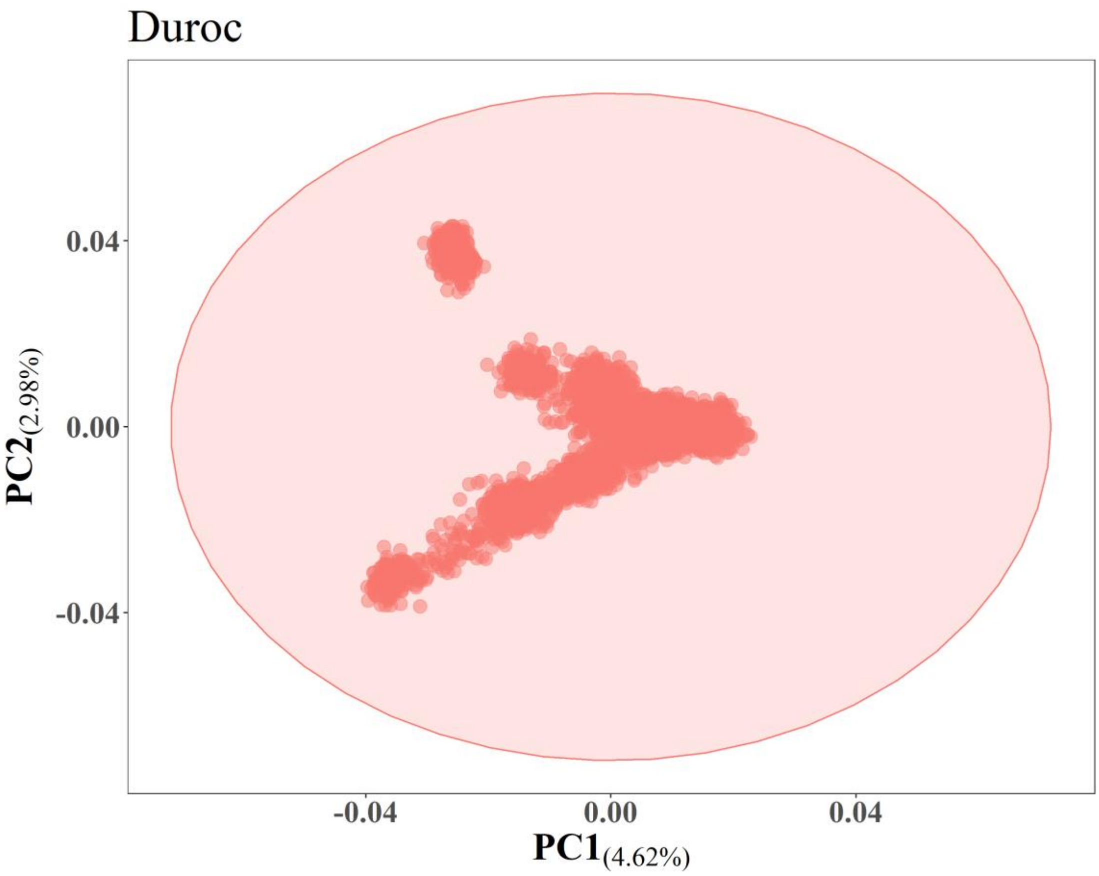

Additional file 1: Figure S1b. PCA plot of Yorkshire.

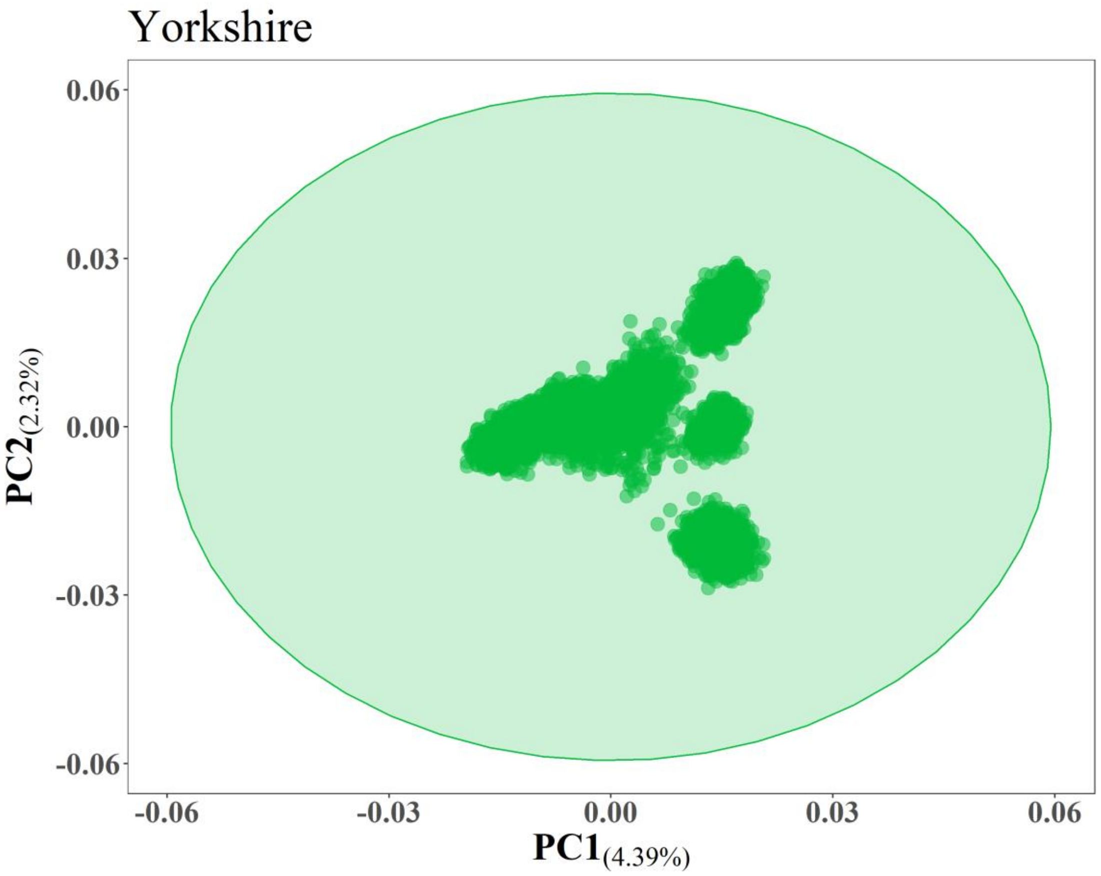

Additional file 1: Figure S1c. PCA plot of Landrace.

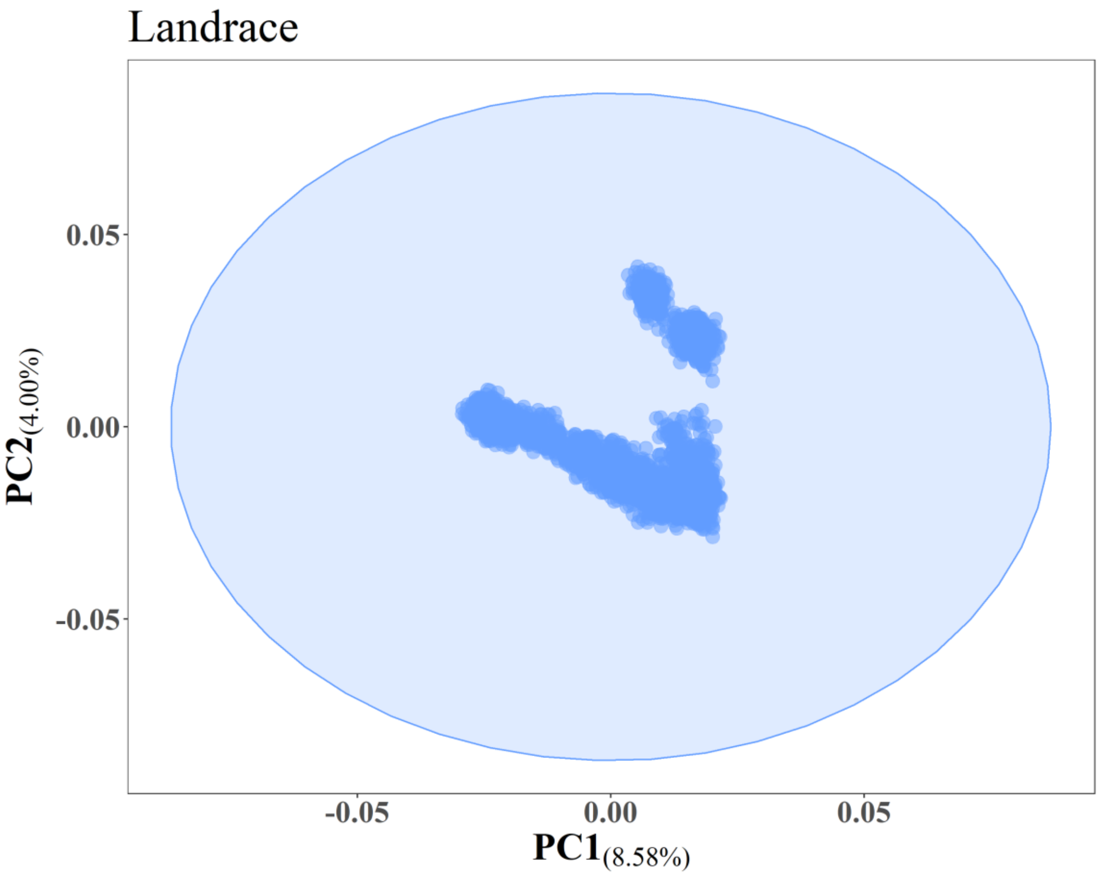

Supplement: Supplementary file 1 — Additional file 1: Figure S1. PCA plot of each of three breeds. [file 12864_2022_9036_MOESM1_ESM.pdf]
