# Supplementary material for: Meta-analysis of genome-wide association studies uncovers shared candidate genes across breeds for pig fatness trait
Source: BMC Genomics. 2022 Nov 30;23:786. doi: 10.1186/s12864-022-09036-z (PMC9714057; doi:10.1186/s12864-022-09036-z)

Additional file 3: Figure S2. Venn diagram of the number of significant SNPs (P<10^{-5}) between 19 single population GWAS and four metaGWAS.

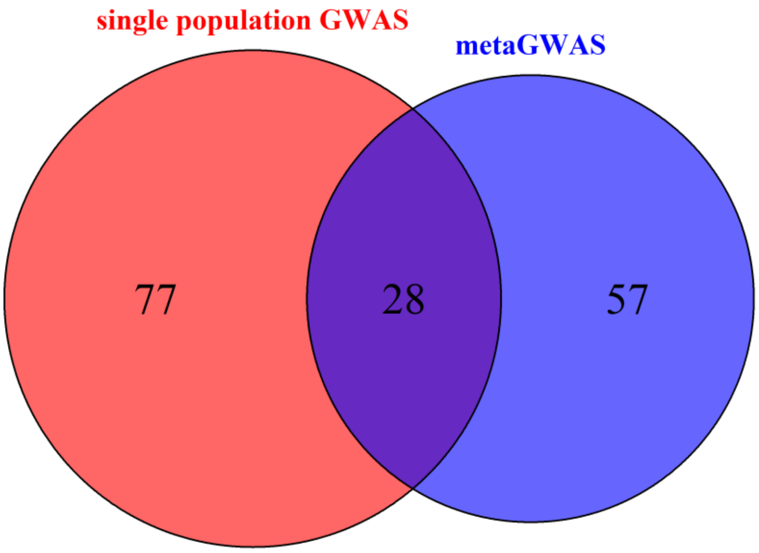

Supplement: Supplementary file 3 — Additional file 3: Figure S2. Venn diagram of the number of significant SNPs (P<10-5) between 19 single population GWAS and four metaGWAS. [file 12864_2022_9036_MOESM3_ESM.pdf]
