# Supplementary material for: Meta-analysis of genome-wide association studies uncovers shared candidate genes across breeds for pig fatness trait
Source: BMC Genomics. 2022 Nov 30;23:786. doi: 10.1186/s12864-022-09036-z (PMC9714057; doi:10.1186/s12864-022-09036-z)

Additional file 5: Figure S3a. Q-Q plot of metaGWAS for Duroc.

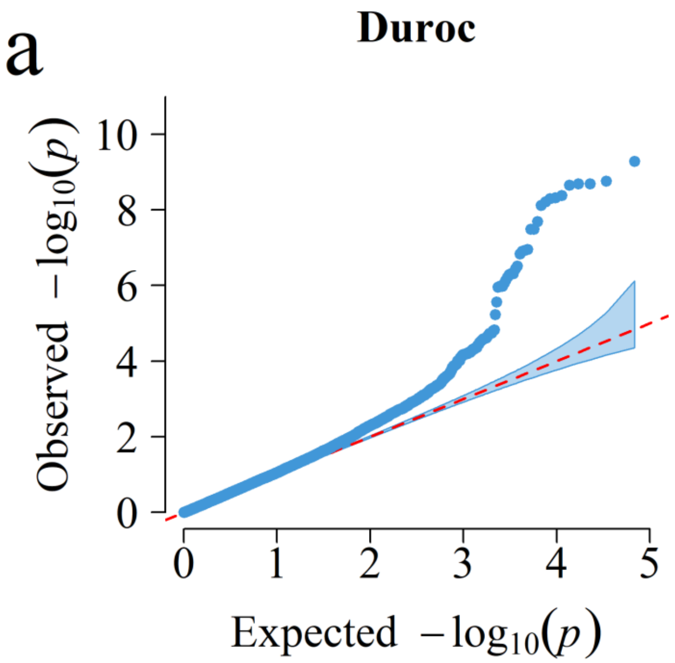

Additional file 5: Figure S3b. Q-Q plot of metaGWAS for Yorkshire.

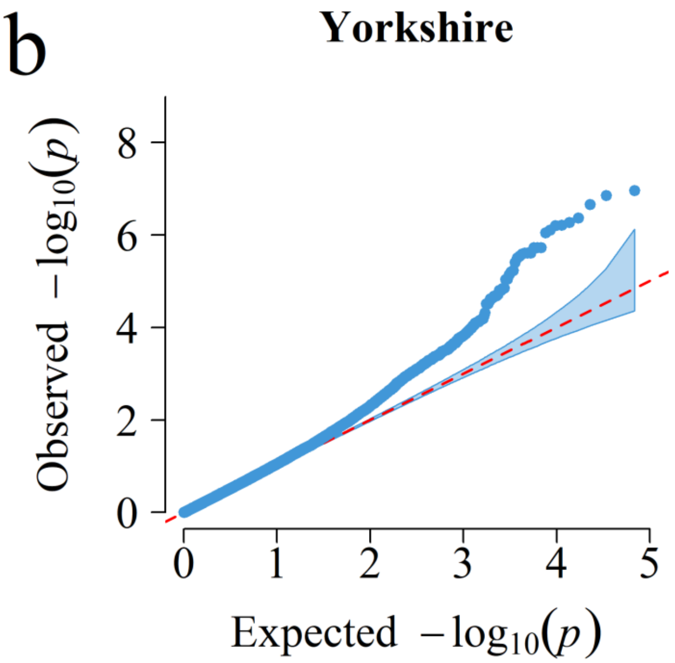

Additional file 5: Figure S3c. Q-Q plot of metaGWAS for Landrace.

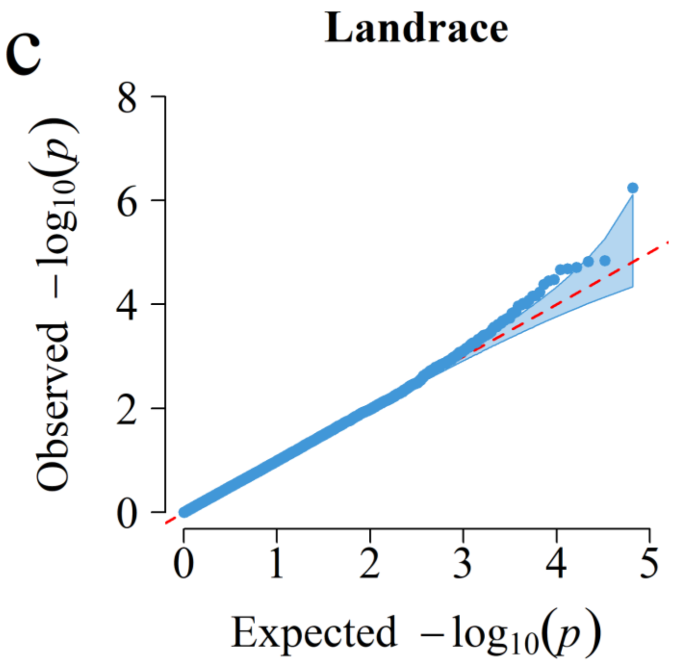

Additional file 5: Figure S3d. Q-Q plot of metaGWAS for across-breed strategy.

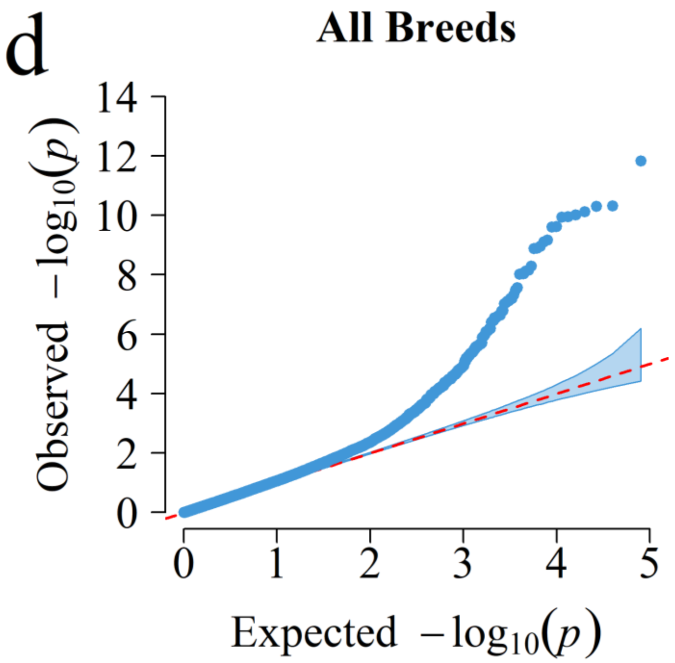

Supplement: Supplementary file 5 — Additional file 5: Figure S3. Q-Q plot of four metaGWAS. [file 12864_2022_9036_MOESM5_ESM.pdf]
