# Supplementary material for: Meta-analysis of genome-wide association studies uncovers shared candidate genes across breeds for pig fatness trait
Source: BMC Genomics. 2022 Nov 30;23:786. doi: 10.1186/s12864-022-09036-z (PMC9714057; doi:10.1186/s12864-022-09036-z)

Additional file 6: Figure S4a. LD blocks of QTL (1: 52,666,889~54,666,889) in each breed (the largest population PP1, PP8, PP15).

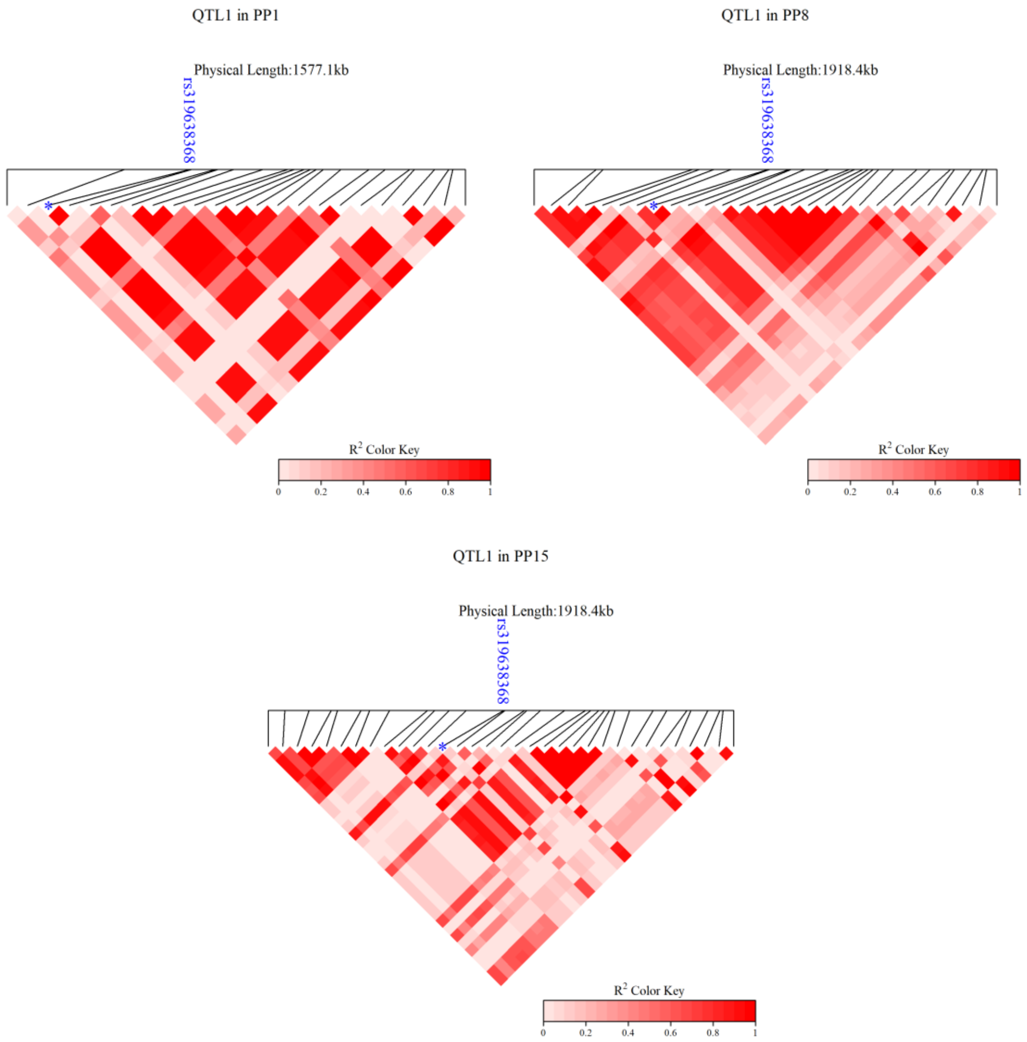

Additional file 6: Figure S4b. LD blocks of QTL (1: 155,986,286~161,824,864) in each breed (the largest population PP1, PP8, PP15).

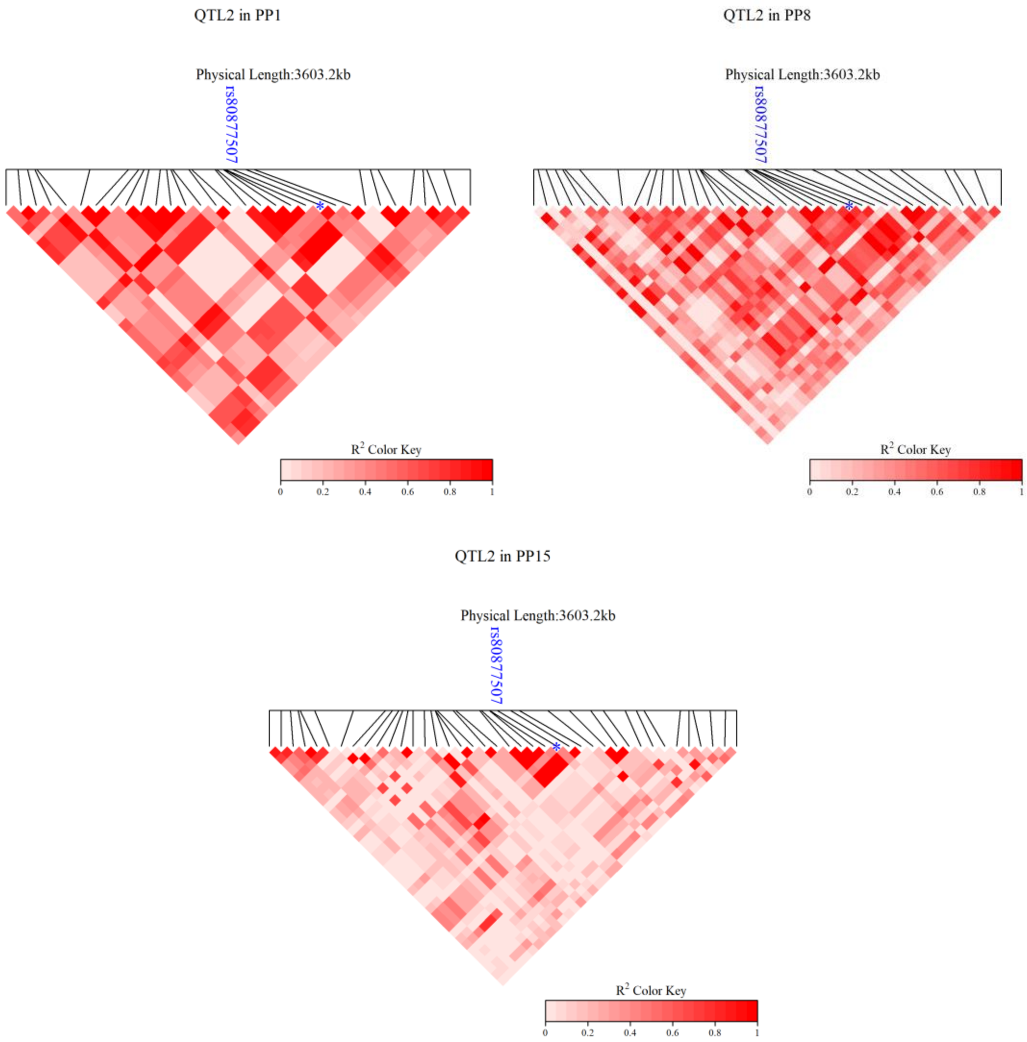

Additional file 6: Figure S4c. LD blocks of QTL (2: 59,697,443~61,697,443) in each breed (the largest population PP1, PP8, PP15).

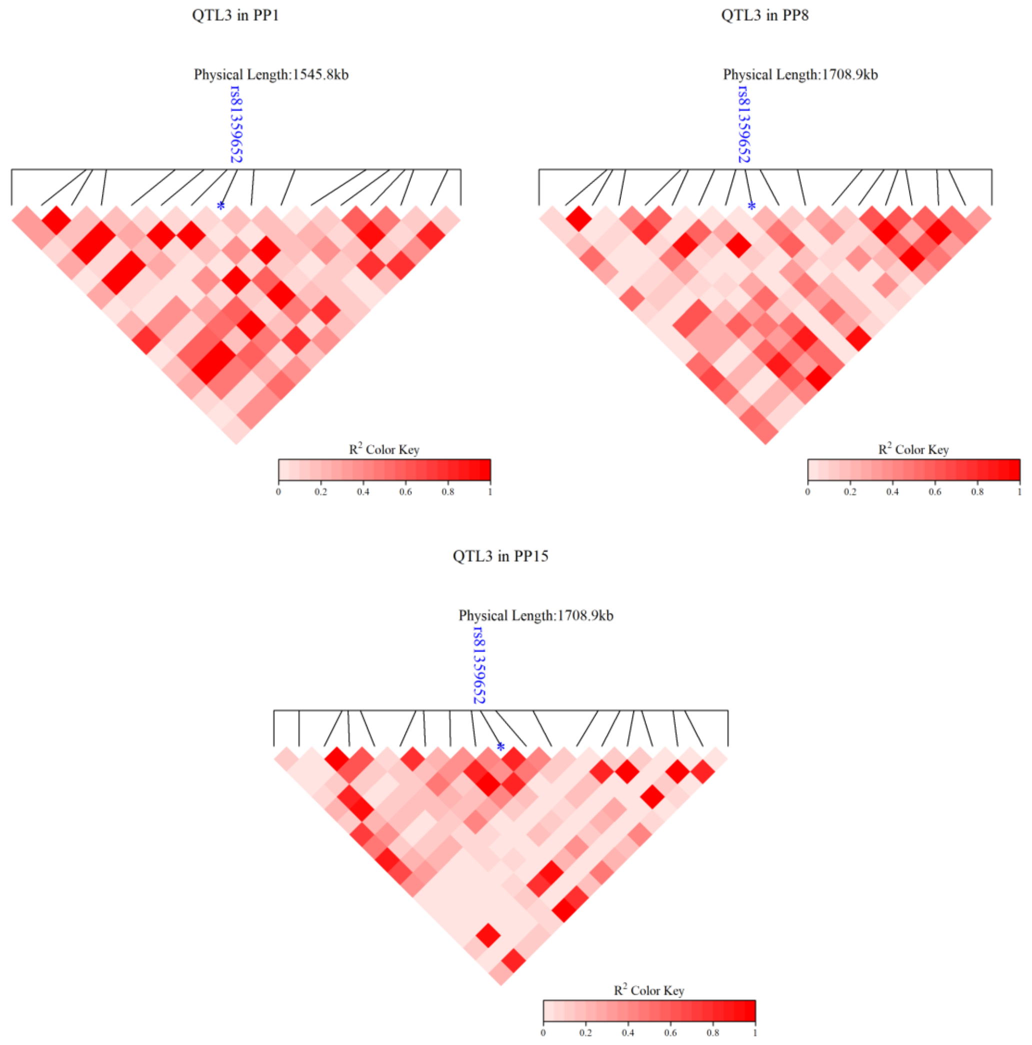

Additional file 6: Figure S4d. LD blocks of QTL (7: 29,476,173~31,569,645) in each breed (the largest population PP1, PP8, PP15).

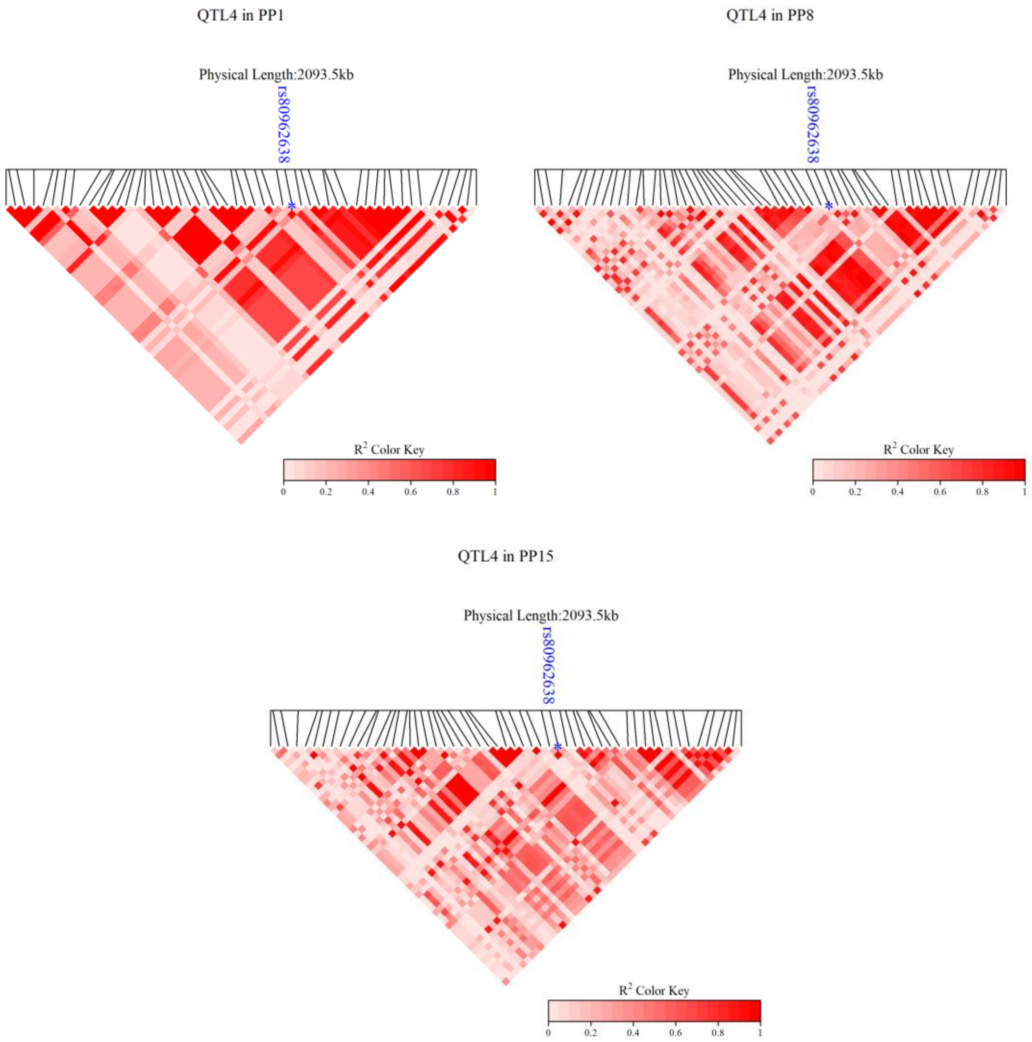

Additional file 6: Figure S4e. LD blocks of QTL (18: 9,555,467~11,555,467) in each breed (the largest population PP1, PP8, PP15).

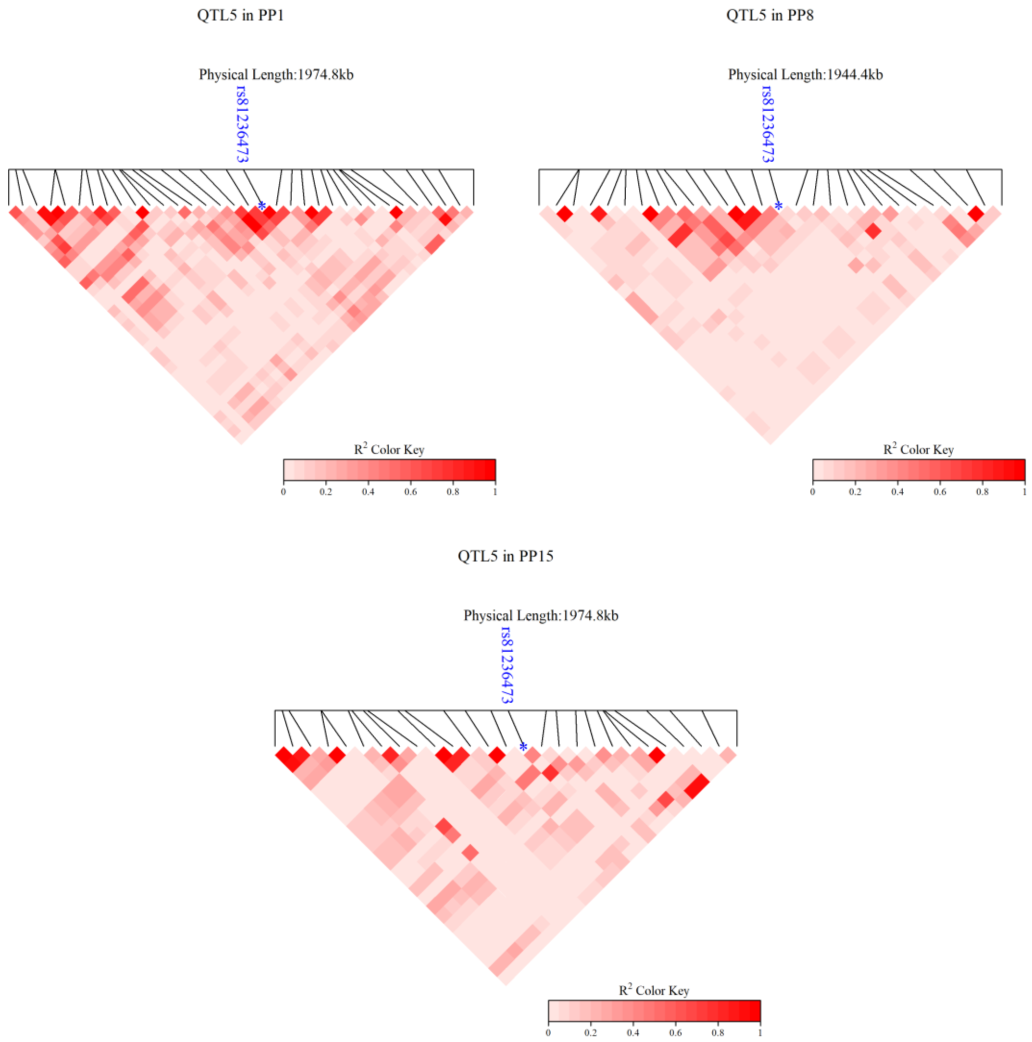

Supplement: Supplementary file 6 — Additional file 6: Figure S4. LD blocks of each of the five QTLs in three breeds. [file 12864_2022_9036_MOESM6_ESM.pdf]
